# Avelumab inducing hypothyroidism and hypoadrenalism: A case report and review of literature

**DOI:** 10.17179/excli2018-1357

**Published:** 2018-06-06

**Authors:** Kashif Aziz, Amir Shahbaz, Muhammad Umair, Isaac Sachmechi

**Affiliations:** 1 Department of Medicine, Icahn School of Medicine Mount Sinai, Queens Hospital Center, Jamaica, New York

**Keywords:** avelumab, hypothyroidism, hypoadrenalism, endocrinopathies, Immune Check Point Inhibitors, Pd-L1

## Abstract

Avelumab is an anti-PD-L1 (programmed death-ligand 1) immune checkpoint inhibitor (ICIs) and the monoclonal antibody that constitutes a major development in the immunotherapy of cancer. In 2017, The European Medicine Agency (EMA) approved it as an orphan drug for treatment of gastric cancer. Avelumab has recently been approved in the United States, Europe and Japan for treatment of metastatic Merkel cell carcinoma (MCC). Avelumab inhibits the interaction of Programmed cell death protein 1 (PD-1) on immune cells with PD-L1 on tumor cells, thus banishing immunosuppressive signals and leading to enhanced immune cell activation. Here we are revealing a case of the patient with metastatic gastric cancer receiving avelumab with the development of undesirable endocrinopathies during the course of treatment. We suggested that patients receiving avelumab immunotherapy should be monitored for signs and symptoms of thyroiditis, hypothyroidism and adrenal insufficiency, which may require immediate attention and supportive treatment by immunosuppression and respective hormone replacement.

## Introduction

Programmed cell death protein 1 (also known as PD-1) is a cell surface receptor that plays a significant role in down-regulating the immune system and promoting self-tolerance by suppressing T cell inflammatory activity. PD-1 is an immune checkpoint and guards against autoimmunity through a dual mechanism of promoting apoptosis in antigen-specific T-cells in lymph nodes while simultaneously reducing apoptosis in regulatory T cells (Francisco et al., 2010[2]; Fife and Pauken, 2011[1]). A new class of drugs that block PD-1, the PD-1 inhibitors, activate the immune system to attack tumors and are therefore used for varying success to treat some types of cancer (Syn et al., 2017[12]). Avelumab is a whole monoclonal antibody of isotype IgG1 that binds to the programmed death ligand 1 (PD-L1) and therefore inhibits binding to its receptor the PD-1. Formation of a PD-1/PD-L1 receptor/ligand complex leads to inhibition of CD8+ T cells, and therefore inhibition of an immune-related reaction. Immunotherapy aims at ceasing this immune blockage by blocking those receptor-ligand pairs (Joseph et al., 2018[6]). The most common serious adverse reactions to avelumab are immune-mediated adverse reactions (irAEs) which includes rash, pneumonitis, hepatitis, colitis, endocrinopathies, and nephritis as well as life-threatening infusion reactions (Hamid et al., 2013[4]). Hereby we present a case of hypothyroidism and adrenal insufficiency induced by Avelumab (PD-L1 inhibitor) in a 69-year- old patient with metastatic gastric cancer.

## Case Report

A 69-year male presented to us with the recurrence of gastric cancer with pancreatic metastasis. He received 6 cycles of Taxotere, Cisplatima, and 5FU (TCF), a TCF chemotherapy regimen which includes Docetaxel, carboplatin, and 5-fluorouracil. He did not show any response to these medications. After that, he entered into a clinical trial with avelumab which is a PD-L1 inhibitor. After three months of starting avelumab, the patient started to complain of resting tachycardia. He had deranged thyroid function tests (TFT) indicating thyrotoxicosis and treated with tapazole 5 mg every other day for resting tachycardia. Subsequently, being 6 weeks on tapazole, his TFT and heart rates improved. Later, on seven months of treatment (15 cycles) the patient was complaining of fatigue, nausea, vomiting, found hypotensive (blood pressure=93/61 mm HG) and hyponatremia. At that time his relevant blood work-up was given in Table 1(Tab. 1).

MRI, abdomen showed adrenal cortical atrophy. After getting the results of his blood tests and imaging, diagnosis of hypothyroidism and adrenal insufficiency were made and the patient was admitted to the hospital and started on hydrocortisone 20 mg in am and 10 mg in pm. Tapazole was stopped initially and later started on levothyroxine 50 mcg which titrated up to 88 mcg. Patient symptoms improved with this treatment.

## Discussion

Immune evasion is an emerging hallmark of cancer, and oncologists have long sought to connect the power of the immune system to treat cancer (Hanahan and Weinberg, 2011[5]). In the last 5 years, inhibition of 2 immune checkpoints, PD-1 and cytotoxic T-lymphocyte-associated protein 4 (CTLA-4), have significantly changed the landscape for immunotherapy. PD-1 is an immune checkpoint receptor expressed on activated T cells. When bound by PD-L1, PD-1 causes T-cell exhaustion and a favorable environment for tumor growth (Topalian et al., 2015[14]). Immune checkpoint inhibitors (ICIs) that block the programmed death 1 axis (PD-L1, PD-1) are important treatment options in various tumor types. Avelumab also functions as an immune checkpoint inhibitor and has recently been approved in the United States, Europe and Japan for the treatment of metastatic Merkel cell carcinoma (MCC) (Shirley, 2018[9]). Common treatment-related adverse events (TRAEs) with anti-PD-L1/PD-1 agents include low-grade fatigue, pruritus and rash. In addition, potentially serious irAEs, such as high-grade pneumonitis or autoimmune-like side effects, occur in a minority of patients (Postow et al., 2015[8]; Spain et al., 2016[11]; Weber et al., 2015[15]). The exact mechanism of PD-L1 induced endocrinopathies is not known. We suggest that cell lytic properties of this immunotherapeutic agent cause thyroiditis, and eventually lead to hypothyroidism and possible adrenalitis leading to hypoadrenalism. In some cases hypophysitis was also property, but not in our patient. On review of the literature, we found 4 studies and trials regarding the use, safety, and immune-related adverse events by the use of avelumab and other immune checkpoints inhibitors. These are shown in Table 2(Tab. 2) (References in Table 2: Kelly et al., 2018[7]; Hahn et al., 2017[3]; Sosa et al., 2018[10]; Sznol et al., 2017[13]) below. 

## Conclusion

Patients receiving avelumab and other PD-1/PD-L1 inhibitors should be monitored for signs and symptoms of immune-mediated adverse events. With the exception of immune-mediated endocrinopathies, most immune-mediated adverse events can be treated with immunosuppression with corticosteroids. For endocrinopathies like hypothyroidism, thyroiditis, adrenal insufficiency and hypophysitis we have to monitor hormone levels and continuous respective hormone replacement.

## Financial interests

None declared.

## Conflict of interest

The authors declare that they have no conflict of interest.

## Figures and Tables

**Table 1:**
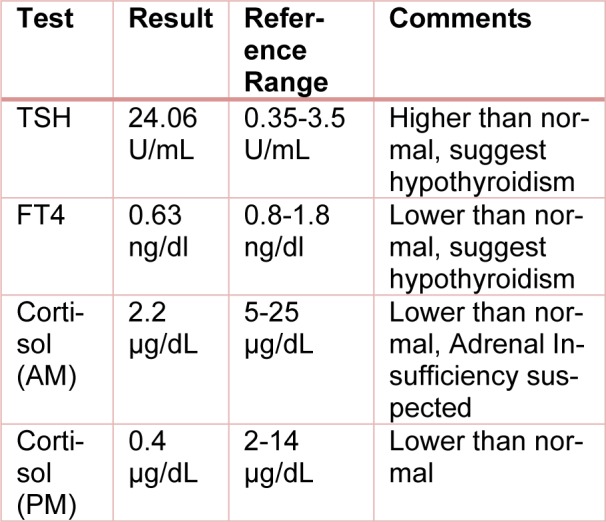
Relevant blood tests and their results

**Table 2:**
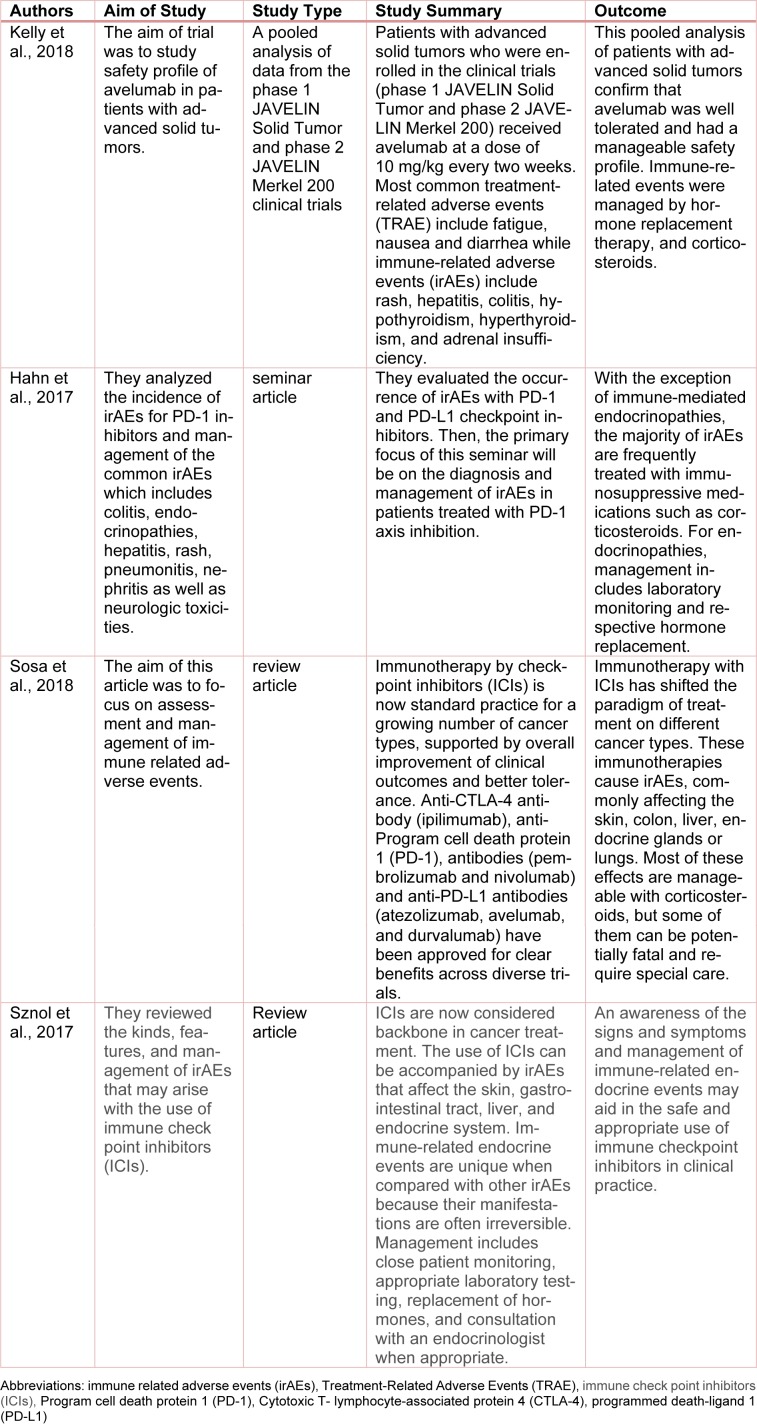
Review of literature regarding the use, safety, and immune related adverse events (irAEs) by the use of avelumab and other immune checkpoint inhibitors
